# NASH/NAFLD-Related Hepatocellular Carcinoma: An Added Burden

**DOI:** 10.3390/life14010025

**Published:** 2023-12-23

**Authors:** Doina Georgescu, Daniel Florin Lighezan, Ciprian Ilie Rosca, Daciana Nistor, Oana Elena Ancusa, Ioana Suceava, Mihaela Adela Iancu, Nilima Rajpal Kundnani

**Affiliations:** 1 Department of Internal Medicine I—Medical Semiotics I, Centre for Advanced Research in Cardiovascular Diseases and Hemostaseology, “Victor Babeș” University of Medicine and Pharmacy, Eftimie Murgu Sq. No. 2, 300041 Timișoara, Romania; 2 Department of Functional Sciences, Physiology, Centre of Imuno-Physiology and Biotechnologies (CIFBIOTEH), “Victor Babes” University of Medicine and Pharmacy, Eftimie Murgu Sq. No. 2, 300041 Timisoara, Romania; 3 Centre for Gene and Cellular Therapies in Cancer, 3000723 Timisoara, Romania; 4 Department 5, Carol Davila University of Medicine and Pharmacy, Dionisie Lupu Street, No. 37, Sector 2, 020021 Bucharest, Romania; 5 Department of Cardiology—Discipline of Internal Medicine and Ambulatory Care, Prevention and Cardiovascular Recovery, “Victor Babeș” University of Medicine and Pharmacy, Eftimie Murgu Sq. No. 2, 300041 Timișoara, Romania; knilima@umft.ro; 6 Research Centre of Timisoara Institute of Cardiovascular Diseases, “Victor Babeș” University of Medicine and Pharmacy, Eftimie Murgu Sq. No. 2, 300041 Timișoara, Romania

**Keywords:** HCC, NASH, COVID-19 pneumonia, obesity, dyslipidemia

## Abstract

Hepatocellular carcinoma (HCC) is the most frequently found primary malignancy of the liver, showing an accelerated upward trend over the past few years and exhibiting an increasing relationship with metabolic syndrome, obesity, dyslipidemia and type 2 diabetes mellitus. The connection between these risk factors and the occurrence of HCC is represented by the occurrence of non-alcoholic fatty liver disease (NAFLD) which later, based on genetic predisposition and various triggers (including the presence of chronic inflammation and changes in the intestinal microbiome), may evolve into HCC. HCC in many cases is diagnosed at an advanced stage and can be an incidental finding. We present such a scenario in the case of a 41-year-old male patient who had mild obesity and mixed dyslipidemia, no family or personal records of digestive pathologies and who recently developed a history of progressive fatigue, dyspepsia and mild upper abdominal discomfort initially thought to be linked to post-COVID syndrome, as the patient had COVID-19 pneumonia a month prior. The abdominal ultrasound revealed a mild hepatomegaly with bright liver aspect of the right lobe (diffuse steatosis), a large zone of focal steatosis (segments IV, III and II) and a left lobe tumoral mass, highly suggestive of malignancy. Point shear wave elastography at the right lobe ruled out an end-stage chronic liver disease. Additional laboratory investigations, imaging studies (magnetic resonance imaging) and histopathological examination of liver fragments confirmed a highly aggressive HCC, with poorly differentiation-G_3_, (T_4,_ N _1_M _0_) and stage IVA, associated with nonalcoholic steatohepatitis (NASH). A sorafenib course of treatment was attempted, but the patient discontinued it due to severe side effects. The subsequent evolution was extremely unfavorable, with rapid degradation, a few episodes of upper digestive bleeding, hepatic insufficiency and mortality in a couple of months. Conclusions: Diagnosis of NASH-related HCC is either an accidental finding or is diagnosed at an advanced stage. In order to earn time for a proper treatment, it becomes important to diagnose it at an early stage, for which regular check-ups should be performed in groups having the risk factors related to it. Patients suffering from obesity and mixed dyslipidemia should undergo periodic abdominal ultrasound examinations. This should be emphasized even more in the cases showing NASH. Complaints of any kind post-COVID-19 should be dealt with keenly as little is yet known about its virulence and its long-term side effects.

## 1. Introduction

Hepatocellular carcinoma (HCC) is one of the most commonly found cancers of the liver, with an increasing incidence over the past few years. It is quite fatal and is often associated with chronic liver diseases, end-stages fibrosis and cirrhosis. However, in up to 25% of the cases, neither underlying chronic liver pathologies nor cirrhosis are documented [1,2]. In Asia and Africa, a higher incidence of HCC is commonly found to be linked to endemic viral B and C hepatitis, while in Western countries the HCC is often related to metabolic syndrome, obesity, type 2 diabetes mellitus (T2DM) and dyslipidemia. The triggering of the events that link obesity to the occurrence of HCC appears to be initiated by the consumption of increased amounts of simple carbohydrates, an aspect that seems to be confirmed by the fact that limiting unhealthy eating habits might result in a decrease the incidence of HCC in obese patients [3,4,5,6]. The association between an increased body mass (overweight or obesity) and NAFLD was also observed in children. It was also found that the presence of NAFLD is associated with increased intestinal levels of bacterial endotoxin, lipopolysaccharide-binding protein and inflammation markers such as tumor necrosis factor (TNF)-alpha and interleukin (IL)-6; furthermore, improving these aspects after a period of obesity treatment can have positive outcomes [7].

Regarding dietary regimes limited to the intake of some nutrients in excess to the detriment of others (such as ketogenic or high protein diets), even if they show a temporary benefit on weight management, the negative effects on liver metabolism cannot indicate them as a solution to fight obesity [8]. Many studies have concluded that obesity is an independent risk factor for the development of HCC and is associated with an increased mortality. Based on observational studies, it can be stated that a 5-unit increase in the body mass index (BMI) can increase the risk of developing liver malignancy by approximately 39% [9,10,11], while several studies have reported the existence of a close link between the nonalcoholic steatohepatitis (NASH)-related cirrhosis and HCC, the latter of which can be also linked to noncirrhotic nonalcoholic fatty liver disease (NAFLD) [12,13,14,15]. However, the risk of developing HCC related to NAFLD in noncirrhotic patients seems to be relatively low [16,17].

The evolution of liver changes from NAFLD to the appearance of HCC comprises of several stages [8]. Passing through the stage of NASH that will later evolve to the stage of liver cirrhosis represents mandatory evolutionary stages of this pathophysiological chain, being also the strongest risk factor for closing the evolutionary chain of the occurrence of HCC in approximately 10% of patients with NAFLD [8]. T2DM, a metabolic disorder characterized by impairment of carbohydrate metabolism with an increasing incidence worldwide, is also linked to the risk of HCC with NAFLD as a starting point [18,19]. Given the extremely frequent association between T2DM and NAFLD, it remains unclear which of the two entities precedes the other [20,21]. The existing inflammation both in patients with NAFLD and T2DM as well as in those with HCC makes the idea that this would be the major triggering element of the neoplastic transformation of liver disease very plausible [8]. Cytokines that are frequently elevated include TNF-alpha, interleukins (IL-8, IL-13) and chemokine ligands (CCL-3, CCL-4, CCL-5, and C-reactive protein [22,23,24].

Another possible mechanism increasingly incriminated by recent research is the dysbiosis of the intestinal microbiome. Xiang Zhang et al. demonstrated that the distinct changes that occur in the intestinal microbiota of patients with HCC are triggered by increased dietary intake of cholesterol [25]. At the level of the intestinal microbiome *Mucispirillum*, *Desulfovibrio*, *Anaerotruncus* and *Desulfovibrionaceae* increase sequentially; while the *Bifidobacterium* and *Bacteroides* concentrations deplete [25]. Administration of atorvastatin is believed to restore the dysbiosis produced by high cholesterol intake and further prevents the high cholesterol intake in NAFLD/HCC-related issues [25]. Probiotics given with atorvastatin further help in improving NAFLD by not only improving the intestinal microbiome but also maintaining serum lipid concentrations and total bile acids [26]. Matsui et al. demonstrated that ileal bile acid transporter inhibitor is capable of ameliorating obesity and NAFLD by improving the gut microbiome [27]. Nuciferin can intervene in the intestinal metabolism of bile acids, further restoring the intestinal dysbiosis and eventually decreasing severity of NAFLD [28]. Also, with the use of an extract of Penthorum chinese Pursh., Gansukeli, Xiaoxi Li et al. demonstrated an improvement in intestinal microbiota in patients with a high dietary intake of lipids, thus intervening on the evolution of NAFLD [29]. The increased dietary intake of quinoa in NAFLD patients renders a positive impact on the composition of the intestinal microbiome, resulting in the improvement and prevention of NAFLD. It further helps reduce the hepatomegaly, splenomegaly and decreases TNFα and IL-10 concentrations, as well as lowers superoxide dismutase and glutathione peroxidase activity [30].

Genetic predisposition and environmental factors related to lifestyle and diet play an important role in the development of HCC in NAFLD/NASH patients. These risk factors could trigger immune-mediated reactions and inflammatory responses, augmenting the oxidative stress and, eventually, DNA alterations [31]. Given the possible evolution of NAFLD/NASH toward HCC in noncirrhotic patients, it has been hypothesized that in these patients hepatic adenomas could at some point turn into malignancy, depending on the exposure to various risk factors [32]. Genetic studies have so far failed to identify the multiple associations of genetic code mutations linking NAFLD–HCC, but single-nucleotide-polymorphisms have been identified as risk factors for the association of HCC with T2DM and NAFLD (e.g., rs738409 polymorphism in phospholipase domain-containing 3, as well as reduced nuclear receptor coactivator 5 expressions are intensified in HCC-T2DM patients) [33,34,35,36,37]. Statistically significant associations between HCC and mutations of genes involved in hepatic lipid metabolism were also reported for the following: patatin-like phospholipase domain-containing protein 3, glucokinase regulator, transmembrane 6-superfamily member 2, membrane bound O-acyltransferase domain containing [33,38,39,40,41]. Based on these findings, the non-invasive score for assessing the risk of HCC in NAFLD patients, the polygenic risk score (PRS), was devised with superior accuracy in predicting the risk of developing HCC in NAFLD patients both in individuals with severe fibrosis, as well as in those with or without associated liver cirrhosis (specificity of approximately 90%, but with limited sensitivity), including among Europeans [38,42]. A mutation in 17ß-hydroxysteroid dehydrogenase type 13 was also identified, with a protective effect in the occurrence of HCC in patients with NAFLD, also preventing the onset of severe liver fibrosis [43].

## 2. Case Report

A young male patient presented with recent history of progressive fatigue, dyspepsia and upper abdominal discomfort. The patient had no family history of digestive diseases, nor he had any history of previous H. Pylori infection, systemic diseases or any organic conditions. The patient was non-smoker, non-alcoholic and not a drug addict. No history of having viral hepatitis was found, nor did he travel or work in viral hepatitis endemic zones, nor had he any exposures to toxic substances including aflatoxin. He had no ongoing treatment at the time of presentation. However, his recent history was marked by an episode of COVID-19 pneumonia, confirmed by RT-PCR test and chest CT scan. The CT-scan illustrated a pneumonia situated in the left upper lobe with an associated minor atelectasis due to the narrowing of a sub-segmentary bronchia and prominent multiple mediastinal lymph nodes with a maximal diameter of 19 mm located around the aortic-pulmonary window, left hilum and at the left inferior paratracheal space. The patient was subsequently hospitalized in a COVID-19-support hospital and his clinical outcome was satisfactory. The patient was discharged after ten days of hospitalization having good respiratory recovery (O_2_ saturation = 97%). The initial finding of the mild atelectasis and the location of the pneumonia at the level of the upper lobe had prompted the indication for bronchoscopy, which was performed after the patient was discharged, but no suspicious lesions were reported and therefore no biopsies were taken. The bronchial lavage did not exhibit cellular atypia, abundance of lymphocytes or other abnormalities, and the subsequent microbiological exams did not display any pathological microbial presence. The infection by Koch bacillus was ruled out based on the normal range of IgG antibodies (Ab), (0.3 UI/mL) at the QuantiFERON-TB Gold Plus test.

**Figure 1:**
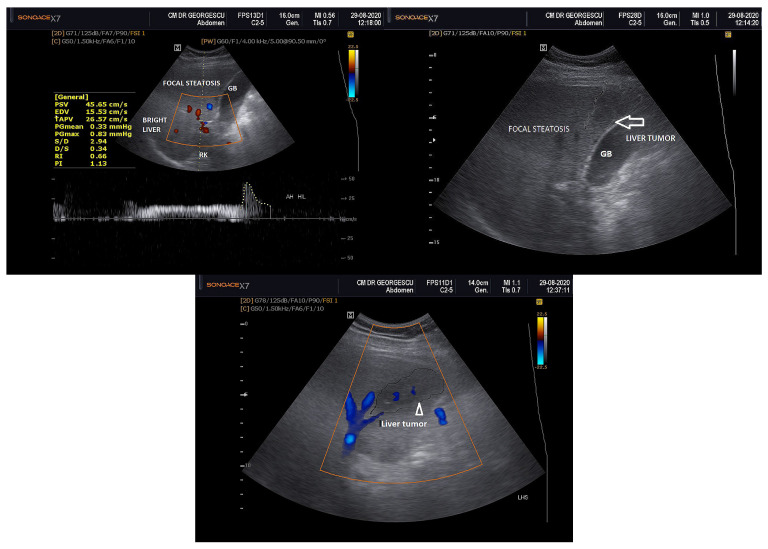
Ultrasound examination of the liver. (**Left**): Triplex exam demonstrates the bright right liver lobe, large focal steatosis, the patency of the portal vein and a mild elevation of the RI of the hilar hepatic artery. (**Right**): 2D exam, arrow pointing at the left lobe’s tumor surrounded by the focal steatosis. (**Middle**): Duplex exam, tip of the arrow pointing at left lobe’s tumor surrounded by the focal steatosis. (GB = gallbladder, RK = right kidney).

Two months after recovery from COVID-19 pneumonia, the patient had a BMI of 33 kg/m^2^, waist circumference of 101 cm, was without any dyspnea and no pulmonary rales were present. Their blood pressure was 125/80 mm Hg and on auscultation no heart murmurs were heard. Their heart rate was 85 beats/min, and the patient had normal diuresis. On abdominal palpation, a mild tenderness in the epigastrium and in the upper right quadrant was noted. Furthermore, moderate hepatomegaly without spleen enlargement was noted, having no signs of portal hypertension, abdominal collateral circulation, jaundice or other liver stigmata. As depicted in Figure 1, a plain transabdominal ultrasonography (US) was performed in fasting state which revealed moderate liver enlargement with shinny, hyperechoic right lobe (segments V–VIII) and a large zone of focal steatosis, near the gallbladder (segment IV) and also at the level of the left lobe (segments II and III). Within this focal steatosis an oval shaped hypoechoic mass, at the level of the left lobe (segments II and III), of 7.3/5.4 cm was noted. It was located in the immediate vicinity of the Glisson’s capsule and extended towards the hilum, it was non-homogeneous and was not well delineated, raising the suspicion of malignancy. The caudate lobe and the spleen had normal sizes and the hilar splenic vein had a normal diameter. The main portal vein had a normal diameter (10 mm), with respiratory variability, hepatoportal flow with clear patency of the main portal trunk and branches, without signs of thrombosis. The hepatic artery at the hilar zone displayed a mild elevation of the resistivity index (RI) = 0.66 at the pulsate Doppler examination. The Morrison’s recess and Douglas pouch presented no transonic liquid accumulation. Perivisceral fat was noted. The liver point shear wave elastography (p SWE), performed at one site of the right lobe, illustrated a median virtual touch quantification (VTQ) of 1.25 m/s, with an estimated fibrosis score of F_0_/F_1_.

Lab work-ups revealed a mild cytolytic syndrome (ALAT = 64UI/dl, ASAT = 58 UI/dL, de Rittis ratio = 0.9), mixed dyslipidemia (total cholesterol = 293 mg/dL, low-density lipoprotein (LDL) = 231 mg/dl and high-density lipoprotein (HDL) = 58 mg/dL, triglycerides = 168 mg/dL), and elevation in C-reactive protein (CRP) = 13.8 mg/L, alpha- fetoprotein (AFP) = 85 ng/mL, CEA = 11.5 ng/mL and CA19-9 = 58 U/mL^3^. The patient’s other laboratory investigations showed normal results, such as the following: hemoglobin = 14.3 g/dL, leukocytes = 6500/mm^3^, platelets = 325,000/dL, gamma-GT = 32 U/L, alkaline phosphatase = 38 U/L, total bilirubin = 0.51 mg/dL, ELFO: total proteins = 6 g/dL, albumins = 60%, alpha1 globulins = 4.5%, alpha2 globulins = 11.5%, beta 1 globulins = 5.6%, beta 2 globulins = 5.5%, gamma globulins = 12.9%; INR = 1.12, fasting plasma glucose = 92 mg/dL, Hb A_1_C = 5.5%, creatinine = 0.7 mg/dL and uric acid = 5.2 mg/dL. HBs antigen (Ag), Delta ag and anti-HCV Ab were absent, plasma iron = 67.1 µg/dL and copper = 82.5 µg/dL, as well as alpha 1 antitrypsin = 1.5 g/L^3^ which was at a normal level and antinuclear antibodies and antimitochondrial antibodies which were within the normal range too. The calculated fatty liver index (FLI) was 83, indicating a high risk for NAFLD. At that point, the diagnosis of NASH was established, as well as tumoral mass of the left liver, obesity and mixed dyslipidemia. The upper and lower digestive endoscopies were absolutely normal, hence eliminating the need of biopsy. The stool’s examination was found to be negative for fecal H Pylori ag and no occult bleeding was noted. The assessment of stool microbiota performed using next generation sequencing (NGS) method revealed a microbiological chart characterized by enterotype 2 (*Prevotella* dominant), a decrease in Shannon’s biodiversity index of 2.5 and a *Firmicutes/Bacteroidetes* (F/B) ratio of 1.3, with modifications of the other bioindicators such as the following: butyrate production was 3, lactate production was 0.2 and acetate/propionate production 6. The mucin degradation was 21.6, *Prevotella/Bacteroidetes* ratio was 3.5 and lipopolysaccharide (LPS)-positive bacteria was 4.056 with increased *Providencia* and *Sutterella* spp. Important changes were noted regarding fiber degrading bacteria with severe decrease in *Ruminoccocus* spp. (0.305) as well as modifications in neuroactive bacteria with an absence of *Lactobacillus brevis* and *paracasei* and a severe decrease in *Oscillabacter* and *Alistipes* spp.

**Figure 2:**
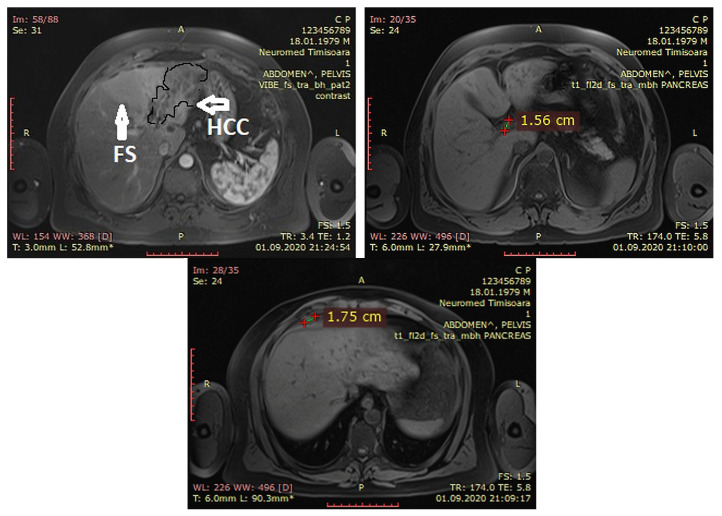
Dynamic contrast-enhanced abdominal MRI. (**Left**): Focal steatosis (vertical arrow) and liver mass of the left lobe (horizontal arrow). (**Right**): Secondary nodules at the segment 1 of the liver. (**Middle**): Secondary nodules at the right supra-diaphragmatic fat. (FS = focal steatosis, HCC = hepatocellular carcinoma).

As seen in Figure 2, the dynamic contrast-enhanced abdominal magnetic resonance imaging (MRI) performed 3 weeks later described the following: a tumoral mass at the left lobe (segments II and III) of 7.8/5.7/7.7 cm, with arterial phase hyperenhancement and washout in the portal venous phase and mild capsular retraction and extension toward the hilar zones were noted. Large focal steatosis were seen on segments II, III and IV. Small, regional lymph nodes with a maximum diameter of 0.7 cm at the level of the hilum were noted. The spleen was within normal range; in the right supradiaphragmatic nodule there was possible secondary lesion in segment I, and a small quantity of ascites without signs of thrombosis of the portal vein and main trunk or branches were noted.

**Figure 3:**
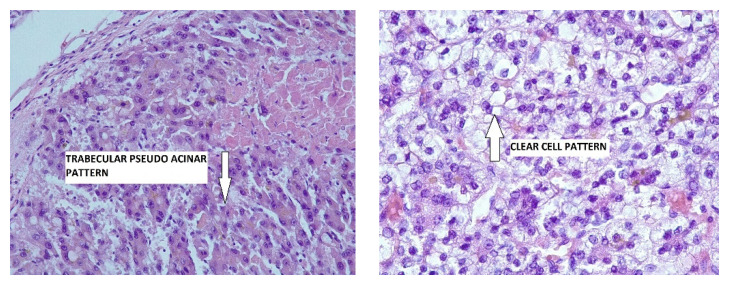
Pathological features of HCC. (**Left**): HCC pseudo glandular pattern (arrow), HE, OM×20. (**Right**): HCC clear cell aspect (arrow), HE, OM×40.

At a later time, the patient was admitted 2 weeks later at the Compartment of Hepato-Biliary Surgery of the General Surgical Clinic, University Hospital, after confirmation of negative RT-PCR during a COVID-19 test. Clinical examination revealed mild scleral jaundice, moderate hepatomegaly and mild-to-moderate ascites without collateral circulation, spider naevi or other signs of liver insufficiency. A diagnostic paracentesis was performed the next day but did not show any atypical cellularity. The serum ascites albumin gradient (SAAG) was 0.9 (<1.1), making the diagnosis of portal hypertension syndrome highly improbable. Laboratory work-ups revealed a mild cytolysis and a minor elevation of the total and direct bilirubin, as well as a mixed dyslipidemia. The brain and thorax helical computer tomography (CT) did not display any secondary metastatic lesions. A few days later an abdominal open surgical intervention was scheduled in an attempt to perform the resection of the liver tumor. The intraoperative findings revealed that the malignancy was in fact more advanced than appeared to be and that the left lobe’s tumor has invaded the surrounding, adjacent tissues, the liver hilum and the hepato-gastric ligament, as well as the peritoneum, leaving no feasible cleavage planes for resection and no other options but taking some tissue biopsies. The liver fragments together with the fragments of falciparum ligament and peritoneum were sent to the pathology laboratory. The immediate postoperative evolution was good under supportive treatment with liver protectors, antalgics, proton pump inhibitors, diuretics and prophylactic antibiotics. The patient was discharged after 10 days of hospitalization in a stable condition with a diagnosis of HCC, T_4_N_1_M_0_, stage IVA, according to the American Joint Committee of Cancer (AJCC), and tumor/node/metastasis (TNM); they were referred to the oncologist. The tissues fragments were analyzed after fixation with 10% formalin and embedding in melted paraffin. The obtained sections were first stained with the usual hematoxylin-eosin (HE) method, with consecutive examination by optical microscopy (OM). As presented in Figure 3, malignant hepatocytes presented a nest-shaped pattern of growth that created trabecular, pseudo glandular features with oversized cells, some of them with an abundant, pale and clear cytoplasm, scattered bilirubin pigment foci and vesicular large nuclei, with coarse chromatin and 1–2 nucleoli. In order to make the distinction of HCC from the metastatic carcinoma and intrahepatic cholangiocarcinoma several immunohistochemical (IHC) special stains were performed, revealing that tumoral cells were IHC positive for cytokeratin (CK) 7 and CK 19, as well as for hepatocyte paraffin antigen (Hep Par)-1 but negative for CK 20. The pathological features were therefore considered consistent with the diagnosis of poorly differentiated HCC with G_3_ grading.

The oncologist’s decision was to put the patient under a course of sorafenib and under supportive ambulatory treatment with liver protectors, vitamins, antiemetics, antiacids, antalgics and diuretics. The patient’s compliance to the treatment with sorafenib was very low and he decided to discontinue it after several weeks due to oral ulcers and the severe digestive side effects. At this point, the patient, and his family also, refused an alternative oncological plan. The further evolution was extremely unfavorable; a rapid degradation developed within a couple of weeks with severe nausea and anorexia, occasional vomiting, weight loss and muscles waste, large ascites, pitting ankle edema and liver insufficiency with intense jaundice and hepatic encephalopathy. Given the pandemic situation and difficulties in travelling, the last visits were arranged via telemedicine and no further imaging studies or other tests were performed. Later the patient had suffered a few episodes of upper digestive bleeding, treated by parenteral esomeprazole and followed by oral lactulose and rifaximin. Eventually, the patient went into a coma and died at the age of 41 years. No postmortem examination was performed due to the ongoing pandemic crisis at that time.

## 3. Discussions

The risk factors for HCC development have been widely studied over the past years. Apart from infections by hepatitis B virus (HBV) or hepatitis C virus (HCV), known for their mutagenic abilities, and exposure to toxins such as alcohol or aflatoxin from contaminated food due to improper storage, there is growing evidence that obesity, T2DM and NAFLD represent increasingly important risk factors for HCC, especially in patients with end-stage chronic liver disease [32,44]. Many possible pathways have been involved in NAFLD-related HCC development. It seems that patients with NAFLD and metabolic syndrome display a long-term systemic inflammatory condition, associated with lipotoxicity, gut microbiota dysbiosis with endotoxinemia and augmented LPSs and microbiota-associated molecular patterns (MAMPs) [45]. Obesity is considered as an independent risk factor for HCC development and obese patients display a 1.93-fold higher risk of developing a primary liver cancer. There is sufficient evidence to prove that leptin, a cytokine which has profibrotic and proangiogenic capabilities, could trigger the proinflammatory cascade, via Janus kinase activation in obese patients with excessive perivisceral fat. It has been also observed that obesity is associated with low levels of adiponectin and anti-inflammatory cytokines, as well as with insulin resistance and excessive fat liver storage [46].

This case report highlighted the possibility of late diagnosis of NASH/NAFLD-related HCC in obese patients without cirrhosis, a condition associated with higher burden and poorly evolution, as others have previously reported. Diagnosis in advanced stages of malignancy is partly a result of inconsistent follow-up in NAFLD patients for HCC early diagnostic in comparison to rest of patients infected by viral hepatitis or having an alcoholic liver pathology [47].

Over the past decades, gut microbiota dysbiosis has been increasingly associated with various conditions, either functional, such as irritable bowel syndrome [48,49] or organic such as atherosclerosis and hypertension [50,51,52] as well as metabolic syndrome and NAFLD [53,54]. There is growing support for the idea that gut microbiota dysbiosis could contribute to NAFLD development, with numerous pathways being engaged. Dysregulation of energy metabolism with lipid build-up as a consequence of hepatocytes de novo lipogenesis and imbalance of very-low-density of lipoproteins (VLDL) with a decreased export of the lipids from the liver and leaky gut with elevation of LPSs ultimately triggers the pro-inflammatory cytokines, via Toll-like receptor (TLR)4 are some of the underlying mechanisms proposed in NAFLD [55,56].

Could gut microbiota dysbiosis be related not only to NAFLD/NASH in obese patients but also to primary carcinogenesis of the liver? Leaky gut, dysbiosis and increased levels of LPSs at the portal vein are frequently taken into discussion when it comes to persistent low grades of liver inflammation. Gut microbiota dysbiosis has been implicated in various mechanisms related to the progression of liver diseases and to liver carcinogenesis as well, while it could regulate complex processes like inflammation, fibrogenesis, immune reactivity, liver injury and regeneration. Various modifications of gut microbiota in patients with chronic liver diseases and HCC have been reported by numerous studies [57]. The patient had a particular gut microbiota with a Prevotella-dominant enterotype and a highly unbalanced F.B ratio with an important increase in Bacteroidetes. Significant modifications of bio indicators and multiple disruptions of the intestinal ecology with increase in LPS-positive bacteria and a decrease in fiber-digesting bacterial spp. are associated with lifestyle and dietary habits, with food high in saturated fat and low in vegetable fiber intake noted. These alterations in the gut microbiota are lately increasingly reported in several hepato-biliary conditions [58,59,60,61,62].

Portal vein thrombosis (PVT) and HCC are closely related, as reported by many studies, although sometimes silent manifestations hidden by the liver disease are expressed by sudden decompensation with ascites and encephalopathy, digestive bleeding or abdominal pain.

The recurrence of multiple episodes of upper digestive bleeding in the patient’s late evolution raises the problem of a malignant PVT, while the surgery and liver pathology describe a highly invasive tumoral profile.

The use of gut microbiome assessments in obese, dyslipidemia, T2DM or metabolic syndrome patients could be a way to prevent the occurrence of HCC. Interventions performed on laboratory mice resulted in the restoration of the gut microbiome and in the disappearance of the risk of HCC occurrence.

Even if most of the time the beneficial effect of changing the lifestyle is trivialized by the patient, it has been shown that reducing body weight (with the achievement of normal weight), performing sports, applying dietary restrictions as well as prohibiting the consumption of alcohol can improve NAFLD, thus further lowering the risk of developing HCC.

Since 2020, an upward trending superimposed on the COVID-19 pandemic was observed in oncological diseases such as hematological malignancies as well as breast and digestive cancer (colorectal carcinoma, primay or metastatic liver cancer, as well as pancreatic cancer). Could viral infection by COVID-19 play a role in this HCC story? Although there is an abundant literature dedicated to oncogenic viruses, there is not yet enough evidence or knowledge about COVID-19 virus’ possible implications in carcinogenesis. However, in recent pandemic years, the oncogenic role of SARS-CoV-2 was heavily studied [63,64,65,66].

The “cytokine storm”, as a hyper immune response in COVID-19 patients secondary to the release of pro-inflammatory cytokines and the persistence of low-grade inflammatory status, may trigger inflammation-induced organ damage, deeming it a tumorigenesis promoter [67].

The hypoxic microenvironment secondary to virus-induced angiotensin-converting enzyme 2 depletion seen in patients with COVID-19 infection is highly linked to oxidative stress and malignant transformation [68]. It seems that the hypoxic microenvironment may also contribute to an increase in tumor cells invasion and may facilitate cell migration and metastasis [69].

Immune dysregulation, with several alterations to the immune mediators and some signaling pathways, shared by COVID-19 infection, is known to be an important step in tumor genesis [70].

As in other RNA viruses, the SARS-CoV-2 RNA genome is integrated into the host chromosomes in a process that may be followed by oncogenesis, depending on activation or deactivation of several oncoproteins and tumor suppressors. Whether this hypothesis can really be demonstrated in SARS-CoV-2 viral infection is still a subject to debate [71].

Considering the high risk for HCC development in patients with NAFLD, a protocol for surveillance needs to be implemented. Unlike other patients at risk for HCC where algorithms for surveillance are fully stated, to date there is no clear consensus in the follow-up, especially in that of non-cirrhotic NAFLD patients, in order to early diagnose the HCC [72,73,74].

Moreover, given that the most common noninvasive imaging method for the diagnosis of the fatty liver condition remains the ultrasound examination, many difficulties are encountered in reaching an early diagnosis of HCC in this particular risk population [75]. Furthermore, many studies highlighted that surveillance using ultrasound examination and alpha fetoprotein testing is not cost-effective, especially in non-cirrhotic NAFLD patients [74]. Reliable candidate biomarkers and effective risk scores for the early diagnosis of HCC are not yet validated [76].

## 4. Conclusions

Since HCC is one of the commonly found cancers of the liver, and is capable of rapidly deteriorating the patients’ health, early detection becomes important. Obesity is considered one of the major risk factors, and life-style changes and diet can lower the risk of developing HCC. Family physicians and gastroenterologists should keep a close eye on any symptoms that can give a clue to the ongoing malignant process in high-risk people. Since our knowledge is limited regarding the long-term COVID-19 complications and considering the growing body of literature claiming a possible link between SARS-CoV-2 and malignancies, periodic monitoring of patients is crucial. In particular, patients that shows the presence of risk factors such as NASH or NAFLD can help in early detection, further earning time to reverse some of the reversible risk factors or begin rapid initiation of proper treatment plans to slow the process of disease.

## Data Availability

Data will be provided upon written request.
